# Application of Neonatologist Performed Echocardiography in the Assessment and Management of Neonatal Heart Failure unrelated to Congenital Heart Disease

**DOI:** 10.1038/s41390-018-0075-z

**Published:** 2018-08-02

**Authors:** Philip T. Levy, Cecile Tissot, Beate Horsberg Eriksen, Eirik Nestaas, Sheryle Rogerson, Patrick J. McNamara, Afif El-Khuffash, Willem P. de Boode, on behalf of the European Special Interest Group ‘Neonatologist Performed Echocardiography’ (NPE), T. Austin, K. Bohlin, M. C. Bravo, C. R. Breatnach, M. Breindahl, E. Dempsey, A. M. Groves, S. Gupta, Z. Molnar, C. C. Roehr, M. Savoia, U. Schubert, C. E. Schwarz, A. Sehgal, Y. Singh, M. G. Slieker, R. van der Lee, D. van Laere, B. van Overmeire, L. van Wyk

**Affiliations:** 1 Department of Pediatrics, Washington University School of Medicine, Saint Louis, MO USA; 2 Department of Pediatrics, Goryeb Children’s Hospital, Morristown, NJ USA; 3 Department of Pediatrics, Clinique des Grangettes, Chêne Bougeries, Switzerland; 4 Department of Pediatrics, Møre and Romsdal Hospital Trust, Ålesund, Norway; 5 Institute of Clinical Medicine, Faculty of Medicine, University of, Oslo, Norway; 6 Department of Cardiology and Center for Cardiological Innovation, Oslo University Hospital, Rikshospitalet, Oslo, Norway; 7 Department of Paediatrics, Vestfold Hospital Trust, Tønsberg, Norway; 8 The Royal Women′s Hospital, Parkville, VIC Australia; 9 Departments of Pediatrics and Physiology, University of Toronto, Toronto, ON Canada; 10 Department of Neonatology, The Rotunda Hospital, Dublin, Ireland; 11 Department of Pediatrics, The Royal College of Surgeons in Ireland, Dublin, Ireland; 12 Department of Neonatology, Radboud University Medical Center, Radboud Institute for Health Sciences, Amalia Children’s Hospital, Nijmegen, The Netherlands; 13 Department of Neonatology, Rosie Hospital, Cambridge University Hospitals NHS Foundation Trust, Cambridge, United Kingdom; 14 Department of Neonatology, Karolinska University Hospital, Karolinska Institutet, Stockholm, Sweden; 15 Department of Neonatology, La Paz University Hospital, Madrid, Spain; 16 Department of Neonatology, The Rotunda Hospital, Dublin, Ireland; 17 Karolinska University Hospital, Karolinska Institutet, Stockholm, Sweden; 18 INFANT Centre, Cork University Maternity Hospital, University College, Cork, Ireland; 19 Division of Newborn Medicine, Mount Sinai Kravis Children’s Hospital, New York, NY USA; 20 University Hospital of North Tees, Durham University, Stockton-on-Tees, United Kingdom; 21 John Radcliffe Hospital, Oxford, United Kingdom; 22 Department of Paediatrics, University of Oxford, John Radcliffe Hospital, Oxford, United Kingdom; 23 Azienda Ospedaliero-Universitaria S. Maria della Misericordia, Udine, Italy; 24 Department of Clinical Science, Intervention and Technology, Karolinska Institutet, Stockholm, Sweden; 25 Department of Neonatology, University Children’s Hospital of Tübingen, Tübingen, Germany; 26 Department of Pediatrics, Monash University, Melbourne, Australia; 27 Addenbrooke′s Hospital, Cambridge University Hospitals NHS Foundation Trust, Cambridge, United Kingdom; 28 Department of Paediatric Cardiology, Radboudumc Amalia Children’s Hospital, Nijmegen, The Netherlands; 29 Department of Neonatology, Radboud university medical center, Radboud Institute for Health Sciences, Amalia Children’s Hospital, Nijmegen, The Netherlands; 30 Department of Pediatrics, Antwerp University Hospital UZA, Edegem, Belgium; 31 Department of Neonatology, University Hospital Brussels, Brussels, Belgium; 32 Department of Paediatrics & Child Health, University of Stellenbosch, Cape Town, South Africa

## Abstract

Neonatal heart failure (HF) is a progressive disease caused by cardiovascular and non-cardiovascular abnormalities. The most common cause of neonatal HF is structural congenital heart disease, while neonatal cardiomyopathy represents the most common cause of HF in infants with a structurally normal heart. Neonatal cardiomyopathy is a group of diseases manifesting with various morphological and functional phenotypes that affect the heart muscle and alter cardiac performance at, or soon after birth. The clinical presentation of neonates with cardiomyopathy is varied, as are the possible causes of the condition and the severity of disease presentation. Echocardiography is the selected method of choice for diagnostic evaluation, follow-up and analysis of treatment results for cardiomyopathies in neonates. Advances in neonatal echocardiography now permit a more comprehensive assessment of cardiac performance that could not be previously achieved with conventional imaging. In this review, we discuss the current and emerging echocardiographic techniques that aid in the correct diagnostic and pathophysiological assessment of some of the most common etiologies of HF that occur in neonates with a structurally normal heart and acquired cardiomyopathy and we provide recommendations for using these techniques to optimize the management of neonate with HF.

## Introduction

Neonatal heart failure (HF) is a progressive clinical and pathophysiological disease caused by cardiovascular and non-cardiovascular abnormalities that results from impairment of the ventricle to fill with or eject blood properly.^1^ The most common cause of neonatal HF is structural congenital heart disease.^1^ In infants with a structurally normal heart, the most common cause of HF is neonatal cardiomyopathy.^2,3^ Neonatal cardiomyopathy is a diverse group of diseases with various morphological and functional phenotypes that affect the heart muscle and alter cardiac performance in neonates.^4^ While the incidence of cardiomyopathy in children is approximately 1 per 100,000,^2,3,5,6^ the neonatal presentation of cardiomyopathies is relatively less common, but carries a significantly worse prognosis with high cardiovascular morbidity and mortality.^7^

The clinical presentation of neonatal cardiomyopathy is varied, and the diagnostic evaluation of HF is complicated by a wide spectrum of rare genetic causes, numerous acquired causes, and varied clinical presentations that range from asymptomatic to congestive heart failure, multi-organ shock, arrhythmia, or encephalopathy.^4^ A high index of suspicion of cardiomyopathy in the immediate postnatal period and its consequences is important, particularly in infants of mothers with diabetes (IDM), sick neonates with hypoxic ischemic encephalopathy, fetal and neonatal arrhythmias, twin-twin transfusion syndrome, and myocarditis. In these neonates at risk for acquired cardiomyopathy, the integration of clinical and echocardiographic features allows for a better assessment of both immediate risk and long-term prognosis. The echocardiographic assessment of intrinsic abnormality of myocardial systolic function (contractility), diastolic function (relaxation and compliance), and structural growth (hypertrophy and dilatation) can help identify cardiovascular compromise earlier, guide therapeutic intervention, monitor treatment response, and hopefully improve overall outcome.^8^

Neonatologist Performed Echocardiography (NPE) plays a pivotal role in detecting the disease and understanding its’ pathophysiology in neonates. In this review, we discuss the classifications and etiologies of neonatal cardiomyopathies, the most common acquired diseases that manifest with neonatal cardiomyopathies in the immediate postnatal period and the emerging echocardiographic methodology that can help in the correct diagnosis and pathophysiological assessment of neonates with cardiomyopathies.

## Classifications of neonatal heart failure

The appropriate classification of HF in neonates has been hampered by the diversity of its’ etiologies and uncertain pathogenesis.^4^ The causes of HF can be divided into several diverse overlapping categories:

1. Cardiovascular vs. non-cardiovascular (Table 1);

**Table 1:** The common acquired causes of neonatal cardiomyopathy and associated types

CARDIAC	
**Infectious**	Neonatal/ Perinatal Myocarditis (DCM) Sepsis Cardiomyopathy (DCM)
**Hypoxic-Ischemic injury (DCM)**	Perinatal insult (Neonatal encephalopathy) Coronary (Stenosis, in utero compromise, abnormal connection)
**Fetal Volume Overload**	Twin-to-Twin Transfusion (HCM) Severe fetal anemia (DCM)
**Arrhythmogenic**	Paroxysmal fetal SVT (DCM) Incessant atrial or ventricular tachycardia (DCM) Neonatal complete heart block (DCM)
**Maternal Autoimmune Disease**	Neonatal lupus (DCM) Neonatal thyrotoxicosis (DCM) Infant of diabetic mother (HCM)
**Drug Induced**	Steroid therapy (HCM)
NON CARDIAC	
	Renal failure Sepsis

DCM, dilated cardiomyopathy; HCM, hypertrophic cardiomyopathy

2. Pathophysiologic: ventricular pump dysfunction with decreased contractility, or altered preload or afterload with preserved contractility; and

3. Congenital cardiac malformation vs. structurally normal heart (cardiomyopathy; ^1^).

Infants with cardiomyopathy can be further classified as genetic cause, acquired, or mixed.^4^ Regardless of the etiology, the classification of HF severity is based on the Ross Heart Failure Classification, and assigns infants to four different classes (no, mild, moderate, or severe HF) based on feeding history, growth parameters, and physical findings.^9^

The ability to anticipate or screen for HF in infants with congenital cardiac malformation or known genetic causes of cardiomyopathy has significantly improved based on antenatal diagnostic testing capabilities.^10^ However, the infant with a structurally normal heart, who is at risk for an acquired cardiomyopathy, may present in the immediate postnatal period with overt signs of HF and shock that were not expected. For these infants, there must be an even higher index of suspicion to be able to properly assess, manage, and mitigate the HF. In this way, the clinician can screen for the cardiomyopathy with clinical examination and echocardiography at birth based on the known risk factors. The acquired causes of cardiomyopathy that lead to ventricular pump dysfunction may be due to hypoxic ischemic injuries (perinatal insults, coronary anomalies), maternal endocrine disorders (IDM), infectious- or immune-related, fetal/neonatal arrhythmias, fetal preload and afterload alterations (twin-twin transfusion), or iatrogenic drug induced causes (maternal steroid use).

An objective echocardiographic assessment of structure and function is important in classifying the acquired cardiomyopathies into the two most common phenotypic forms in neonates:

1. Congestive/dilated cardiomyopathy (DCM); and

2. Hypertrophic cardiomyopathy (HCM), (Table 2).

**Table 2:** Echocardiographic Features of Cardiomyopathies based on Phenotype

Echocardiographic features	Hypertrophic cardiomyopathy	Dilated cardiomyopathy	Restrictive cardiomyopathy	LV non-compaction
LVEDD	Normal, then decreased	Increased	Normal	Normal, then increased
Atrial size	Increased	Increased	Normal, then increased	Normal, then increased
LV Wall thickness	Increased	Normal	Normal, then increased	Normal, then increased
LV ejection fraction	Normal, then increased	Decreased	Normal, then decreased	Normal, then decreased
RV function	Normal	Normal, then decreased	Normal	Normal, then decreased

LV, left ventricular; LVEDD, left ventricular end diastolic diameter; RV, right ventricular. These phenotypes reflect the underlying chamber size, wall thickness, and ventricular function Hypertrophic and dilated cardiomyopathies are the two most common phenotypes in neonates. The popular classification of cardiomyopathy in older children and adults include three additional major morphological subtypes, (i) Restrictive cardiomyopathy (RCM) (ii) Left ventricular non-compaction cardiomyopathy (LVNC) and (iii) Arrhythmogenic right ventricular cardiomyopathy, (ARVC),^8^ but these present infrequently in neonates, often only with rare genetic related cardiomyopathies.

In HCM, the ventricular walls are hypertrophied, the cavity is small, and initially the ventricular diastolic function is abnormal, sometimes followed by systolic dysfunction.^11^ The structural abnormalities of the myocardium are out of proportion to the afterload of the ventricle.^8^ In DCM, the cavity is enlarged, wall thickness is normal or thin, and ventricular systolic function is primarily depressed. In neonates, HCM and DCM have a similar distribution, with DCM slightly more prevalent.^12,13^

## Echocardiography measures in neonates with heart failure

The diagnostic approach to assess cardiac structure and function in neonates at risk to develop HF is outlined in Table 3.

**Table 3:** Echocardiographic methods of assessing cardiac structure and function in neonates with heart failure

	Measures
**Cardiac Structure**	Volume Linear dimensions of right and left ventricle Left ventricle mass index Relative wall thickness Right ventricle areas
**Left Ventricle Systolic function**	Left ventricular ejection fraction (M-Mode) Left ventricular ejection fraction (Simpson’s Biplane) Left ventricular systolic strain/strain rate Rotational mechanics (apical/basal rotation, LV twist) Systolic mitral annular longitudinal shortening
**Left Ventricle Diastolic function**	Peak transmitral E and A spectral Doppler velocities E/A ratio E deceleration time Pulmonary venous diastolic Doppler velocities Color M mode flow propagation velocities (Vp) Mitral annular tissue Doppler velocities (e‘ & a‘) Mitral AV plane (E/e‘ ratio) LV diastolic strain rate
**Right Ventricle Systolic function**	Tricuspid annular plane systolic excursion Fractional area of change Deformation – longitudinal systolic strain and strain rate
**Right Ventricle Diastolic function**	E/A ratio Diastolic strain rate

## Common diseases that manifest with neonatal cardiomyopathy

### Perinatal hypoxic ischemia

Perinatal hypoxic ischemic insults may result in multi-organ system dysfunction, and are a common cause of acute, often reversible cardiovascular dysfunction in neonates.^14,15^ Although clinicians are most concerned with the hypoxic consequences of the cerebral injury (i.e. neonatal encephalopathy), myocardial failure occurring due to ventricular dysfunction, impaired myocardial contractility, decreased cardiac output, and/or abnormal postnatal circulatory transition may also contribute to the neurological injury and exacerbate organ damage.^16,17^ Myocardial dysfunction has a reported incidence of 30–82% in neonates with severe neonatal encephalopathy,^18–21^ with hemodynamic instability ranging from 33 to 77% in patients receiving therapeutic hypothermia (TH; 15). The incidence of myocardial injury may actually be higher in the preterm infant with neonatal encephalopathy who is already at risk for myocardial dysfunction due to immaturity.^22^ The reduced heart function is often transient, with the most severe cardiovascular abnormalities typically occurring 2 to 3 days after the initial insult, followed by gradual recovery.^23,24^

In cases where the hypoxic ischemic insult causes encephalopathy, term and near-term neonates now receive TH as standard of care.^25^ The cardiac side effects of TH have been documented,^26–33^ but these are outweighed by the benefits on survival and neurodevelopmental outcome.^29^ TH alters systemic and pulmonary hemodynamics through increasing vascular resistance and lowering resting heart rate, which reduces cardiac output.^31,32^ Severe arrhythmia is rare, while transient sinus bradycardia is frequent during the cooling phase of TH, but does not usually require medical treatment.^29^ Cooled neonates have reduced heart function during treatment,^26–28^ probably at a similar level as non-cooled neonates.^28^ Although the heart function is reduced during cooling, the lactate levels that are often high at the start of treatment^28,30^ usually improve during cooling,^28^ due to reduced metabolic demands at low body temperature. The long-term cardiovascular consequences of rewarming and the impact of reperfusion on vital organs are not well delineated.

Although TH has become the standard therapy, the ongoing hemodynamic instability, related either to the primary insult, the effects of TH (cooling and/or rewarming), or even the hemodynamic approach to intervention, may actually decrease the effectiveness of neuroprotective strategies by compromising vital organ perfusion and metabolism.^15,34^ While there is an association between outcome and heart function during TH,^27,35,36^ the long-term cardiac consequences from the perinatal hypoxic ischemic insult are not known. There is also a lack of clarity regarding thresholds for cardiovascular intervention; non-judicious use of inotropic agents may increase the risk of brain injury by promoting excessive cerebral reperfusion.^15^ There is evidence that neonates with higher flow in the superior vena cava are at greatest risk of brain injury.^37^

After the initial perinatal hypoxic insult, myocardial contractility increases and the myocardium works to enhance blood flow and protect against systemic hypoxia.^17,38^ Myocardial injury will ultimately develop when this “compensation mechanism” fails.^39^ Myocardial dysfunction will worsen further following the reperfusion injury due to reactive oxygen species and the injury eventually reduces the contractile responsiveness of the myocardium, causing a significant reduction in cardiac output,^35^ hypotension, and further impairment of cerebral blood flow and perfusion of other organs.^38,40^ The heart may be affected on all levels, but with a predilection for the papillary muscles and sub-endocardial regions.^41–43^ Though there has been no consistent link between the degree of hemodynamic instability and neurodevelopmental outcome,^15^ there are two major patterns of myocardial dysfunction that have been observed in neonates with a perinatal hypoxic insult. The first event results from depression of LV function with subsequent reduced cardiac output. The moderate to severe LV systolic dysfunction may lead to pulmonary venous hypertension because of diastolic impairment.^15^

The second event results from the hypoxia preventing the normal relaxation of the pulmonary vascular bed, and the elevated pulmonary vascular resistance (PVR) causing deoxygenated blood to be shunted to the systemic vasculature.^44^ This persistent pulmonary hypertension (PPHN) picture leads to right ventricular (RV) dysfunction and reduced RV output. As the PPHN worsens (sometimes even after initiation of TH; see Thoresen and Whitelaw, ^31^), the impairment in oxygenation and pulmonary venous return further compounds the already reduced systemic blood flow from LV dysfunction.^14^ TH and rewarming can affect systemic and pulmonary blood pressure.^14,15,38^ If the PPHN worsens it may necessitate an early termination of cooling^31^ with a gradual rewarming may have theoretical benefit in achieving a more controlled change in hemodynamics.^15^ “Rewarming hemodynamics” and how to actively adjust the cardiovascular specific medications throughout the warming period requires further study.^15^ Global ventricular dysfunction, which starts on the left and progresses to the right, may also lead to biventricular enlargement and dilated cardiomyopathy.^7^

#### Cardiovascular Assessment of a Neonate with neonatal encephalopathy

Early and accurate detection of myocardial injury in neonates with neonatal encephalopathy presents an ongoing challenge for the neonatologist. A high index of suspicion for cardiopulmonary dysfunction is important in the neonate with clinical and biochemical evidence of a hypoxic ischemic insult. The clinical signs of myocardial injury and associated PPHN, coupled with the electrocardiographic and radiographic findings are all non-specific and inconclusive.^45^ Cardiac troponins are biochemical markers of myocardial damage, and their levels increase in newborns with neonatal encephalopathy.^23,35,45–53^ Measurement of troponins has replaced the creatine kinase-MB (CK-MB) isoform as the biochemical test for cardiac involvement in neonates with neonatal encephalopathy.^45,46,54^ Troponin levels have prognostic significance with regards to mortality and outcome in perinatal hypoxic ischemia, and elevation of cardiac troponin T and I levels appears to correlate with neurodevelopment at 18 months.^46,55^ Elevation of cord troponin is a good early predictor of severity of encephalopathy from a perinatal hypoxic insult and mortality in term infants.^56^ There may also be a role for serum N-terminal pro-Brain Natriuretic Peptide (NT-pro-BNP) as recent evidence has demonstrated that its levels can reflect myocardial injury in neonates with asphyxia and may also guide diagnosis.^57^

#### Clinical indications for NPE

In the presence of clinical cardiovascular compromise and/or elevation of biomarkers, NPE should be used to first characterize the structural and then functional components of the heart disease. The etiology of an acquired hypoxic-ischemic myocardial insult is either from a perinatal hypoxic event or a coronary insult (stenosis, abnormal origin, or in utero compromise).^7^ In the most severe form, all of these etiologies can result in DCM with depressed global function and performance. If functional abnormalities are detected (with normal structure), NPE may then be utilized to perform longitudinal assessments and serially follow hemodynamic effects of treatment response in the sick neonates with neonatal encephalopathy.^15^ In the absence of clinical compromise or elevated troponin levels, NPE should be considered on an individual basis.^14^ NPE can still be used to provide clinically relevant information, i.e. assess fluid responsiveness and identify an underlying structural contribution to end-organ perfusion. Furthermore, irrespective of blood pressure, cardiac output may not be low, but sometimes quite high with very good myocardial contractility, even during cooling (suggestive of a low SVR). In general, NPE can be used to assess myocardial function, detect PPHN at an early stage, distinguish right-to-left atrial shunting caused by myocardial dysfunction or PPHN, and follow circulatory changes during cooling and rewarming phases of TH.^14^ During the cooling and rewarming phases, the assessment of hemodynamics may be challenging, and there may be a place for the use of NPE in optimizing hemodynamic management.^14,15,38^

#### Echocardiographic assessment techniques

Conventional and emerging quantitative echocardiographic techniques are used to provide clinically useful measures of cardiac performance in the neonate with encephalopathy from a hypoxic ischemic event. Qualitative assessment with NPE in the parasternal long axis and short-axis views can provide useful information about myocardial function but should be considered in conjunction with the clinical examination and other established quantitative measures. For example, visualizing the LV cavity size in end-diastole, may aid in the assessment of fluid responsiveness (hypovolemic neonates will have very little residual cavity in diastole). Although there is a paucity of data on the qualitative assessment of fluid status in neonates using echocardiography, neonates with encephalopathy from a hypoxic ischemic event often receive volume expansion therapy because of a failure to respond to stabilization, rather than clinically suspected hypovolemia. Excessive fluid administration may actually cause further impairment of gas exchange in the setting of myocardial dysfunction.^14^

M-mode function allows for quantification of the initial visual assessment with fractional shortening (FS) and ejection fraction (EF). These conventional measures of LV function assess the changes in cavity dimensions, but may be insufficient to detect overt dysfunction in a timely manner because their measurements are influenced by image quality, inadequate reproducibility and standardization in neonates.^58^ Furthermore, they are both limited in the neonate for assessment of LV systolic function because of the presence of higher RV pressure and reduced septal motion.^58^ Functional assessment by FS often fails to detect impaired heart function in neonates treated at normothermia,^17,45,48,59,60^ especially in milder cases of perinatal hypoxic ischemic insults,^23^ while in more severe cases FS is reduced.^18,27,47,61,62^

Cardiac output can be assessed by measuring the outflow tract diameter and the velocity time integral, and it has been shown that decreased LV outflow and stroke volume is evident in neonates with neonatal encephalopathy.^47^ Although SVC flow is not a true representation of cardiac output, it may potentially act as proxy measure for cerebral blood flow.^14^ The assessment for PPHN should include the various methods of estimation of pulmonary pressure (tricuspid regurgitation velocities and pulmonary artery acceleration times derived pulmonary pressures; ^48,63^), the assessment of patency and direction of shunt in the patent ductus arteriosus, degree of RV enlargement or hypertrophy, right atrial dilation, or any degree of ventricular septal wall flattening. We caution users on the subjective qualitative assessment of RV function, as this method has been shown to be inaccurate.^64^

#### Advances in echocardiographic techniques

**Fig. 1:**
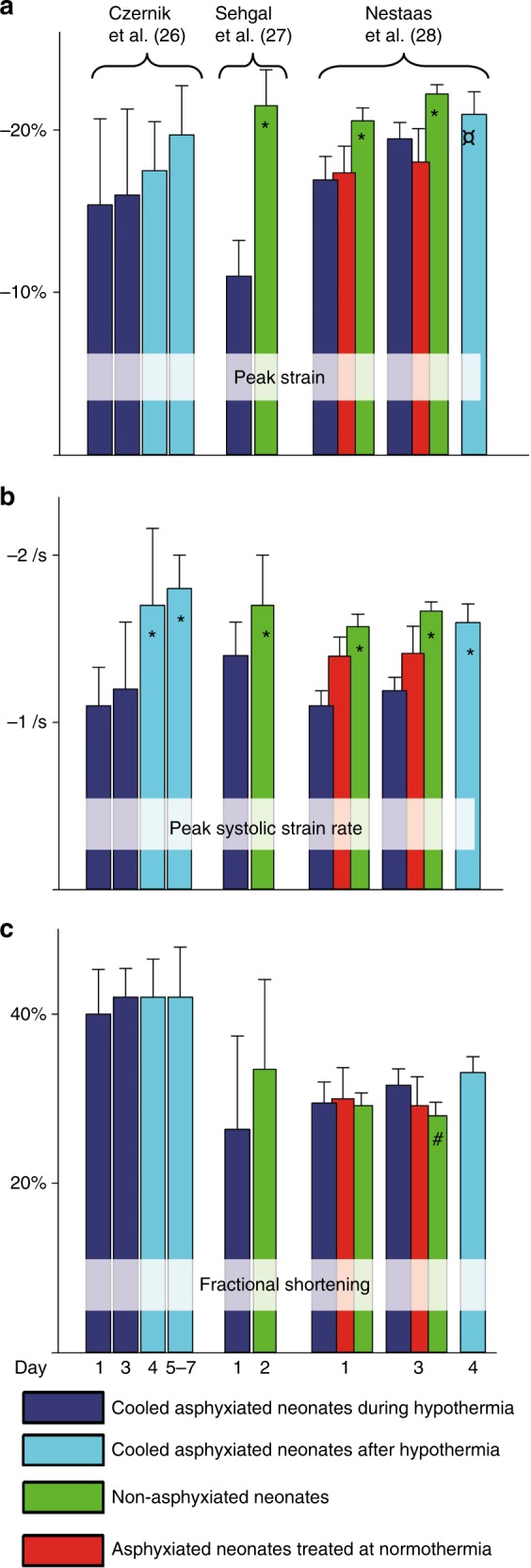
Heart function during and after therapeutic hypothermia demonstrated as (**a**) Peak strain; (**b**) Peak strain rate; and (**c**) fractional shortening. X-axis: Day of life. Y-axis: Heart function indices. Bars are means with 95% confidence intervals. *: Significantly higher than asphyxiated neonates at day 1 and 3. ¤: Significantly higher than asphyxiated neonates on day 3 treated at normothermia. #: Significantly lower than cooled neonates at day 3 and day 4 (The bars for peak systolic strain rate from reference 13 are estimates from segment values). Reprinted with permission.^27^

Advances in neonatal cardiac imaging permit a more comprehensive assessment of myocardial performance in neonatal encephalopathy that could not be previously obtained with conventional imaging. Tissue Doppler (TD) velocities and timing events of the AV-plane,^23,45^ in addition to deformation imaging^24,26^ are more sensitive than FS to detect reduced heart function.^23,45^ Measurements of RV dimensions, RV fractional area change (FAC), tricuspid annular plane systolic excursion (TAPSE) and deformation imaging are feasible for evaluation RV performance (structure and function) in both term and preterm infants^65–67^ and appear to be suitable techniques for measuring changes in myocardial function in neonatal encephalopathy.^26–28,68^ Nestaas et al. demonstrated a decrease in global and regional myocardial function with TD derived strain imaging in infants with neonatal encephalopathy who received TH and normothermic infants. Similarly, Seghal et al.^27^ observed that STE derived strain was impaired and Czernick et al.^26^ showed that strain rate by 2D STE was lower during TH and improved after re-warming, reflective of improvement in myocardial contractility after re-warming. (Fig. 1; ^68^).

### Infants of diabetic mothers

The reversible cardiomyopathy seen in IDM’s is the most common form of HCM during the early neonatal period.^11^ Functional myocardial abnormalities are present in up to 30% of IDMs and include intraventricular septal hypertrophy and cardiomyopathy (HCM). While symptomatic HCM occurs in 10–15% of IDMs, it is found in up to 30% of IDMs when routinely searched for with an echocardiogram.^69,70^ The incidence of HCM is higher in infants born to mothers with pre-gestational DM.^71^ During the gestational periods, the high levels of glucose in the maternal blood will cause fetal hyperglycemia through the placenta and affect the heart in multiple ways, including cardiac malformations, hypertrophic cardiomyopathy, and functional impairment.^72^ IDMs may also suffer from delayed transitional pulmonary circulation that can progress to more severe PPHN.^73^ While most of the alterations in systemic and pulmonary haemodynamics appear to resolve during the first year of age, there is limited information regarding the long-term cardiac performance in the IDM population.

The diagnosis and management of HCM in IDM is based on a thorough understanding of the underlying anatomy and pathophysiology, but the clinical outcome relates directly to the degree of dynamic obstruction to the LV outflow tract (LVOT) and/or to the degree of diastolic dysfunction. IDMs can have abnormal cardiac performance ranging from asymmetrical septal hypertrophy in the mildest cases, non-obstructive HCM in moderate cases, or massive obstructive HCM and major HF in extreme cases. As the most common cardiac pathology in IDMs, asymmetric septal hypertrophy is an anabolic result of fetal hyperinsulinemia triggered by maternal hyperglycemia during the third trimester.^72^ The majority of the IDMs are actually asymptomatic despite this dysfunction, and the septal wall hypertrophy is typically transient, resolving in 2 weeks to 4 months.^74–77^ The myocardial hypertrophy predominantly affects the interventricular septum but may also involve the free walls symmetrically. As the hypertrophy extends to the rest of the myocardium and the right and left posterior walls become thickened and enlarged, the non-obstructive moderate form of HCM leads to mild diastolic dysfunction in the setting of normal systolic function.^78^ In extreme cases of hypertrophy, LVOT obstruction occurs with severe diastolic and systolic dysfunction. The obstructive HCM in the IDM can generate an elevated pressure gradient in the cavity during systole, with maximum peak at the end of the systole, leading to systolic obstruction to LV ejection. The LV mass and contractility are increased and there is LVOT obstruction with apposition of the anterior leaflet of the mitral valve to the interventricular septum during systole, all of which can be seen on echocardiography. The cardiac output is significantly reduced, secondary to reduced stroke volume, and is directly related to the degree of septal hypertrophy.

#### Cardiovascular assessment of an IDM

The approach to cardiovascular care in the IDM should consider actual pathophysiology, phase of intervention, and impact of concomitant treatments. IDMs should be evaluated for clinical signs of:

1. HF (cyanosis, tachypnea, tachycardia, and cardiomegaly on chest radiogram) and

2. PPHN (increase oxygen requirement, increased RV afterload). Dehydration and hyperviscosity (caused by polycythemia) exacerbate these symptoms and should be accounted for in the cardiovascular assessment.

#### Clinical indications for NPE

Echocardiography is used for screening and preclinical diagnosis to detect the different presentations of HCM in IDMs. When clinical signs of HF or PPHN are present, postnatal echocardiography screening and identification of which IDMs to serially follow with NPEs is warranted to modulate infant cardiac dysfunction. In the extreme cases of HCM and PPHN, serial echocardiographic imaging can facilitate the identification of potential candidates for such treatment and help guide therapy. For example, neonatal symptoms of low cardiac output can present with variable severity and could be a result of the diastolic dysfunction (poor ventricular filling) with or without LVOT obstruction or from LV systolic dysfunction. Echocardiograms can follow the response to the decrease in the LVOT gradient and improvement in diastolic function following medical treatment. IDMs with documented septal hypertrophy on echocardiogram and signs of HF or PPHN should be followed.

#### Echocardiographic assessment techniques

**Fig. 2:**
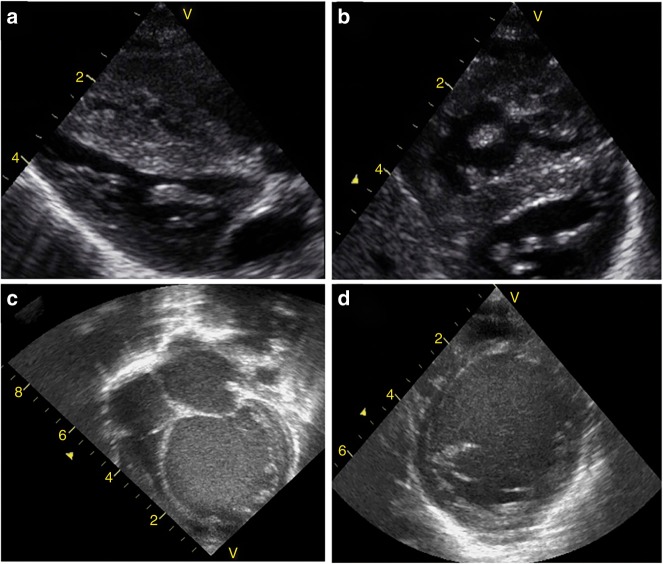
Examples of Echocardiographic findings in common neonatal cardiomyopathies. Infant of diabetic mother: Hypertrophic cardiomyopathy with asymmetric septal hypertrophy. The ventricular walls are hypertrophied, the cavity is small, and ventricular function is normal or hyperkinetic. Two-dimensional echocardiogram showing septal hypertrophy in hypertrophic cardiomyopathy in the parasternal long axis view (Panel a) and the short axis view (Panel b). Arrhythmia-induced neonatal cardiomyopathy (AINC) with severely dilated cardiomyopathy with LV involvement in the apical 4-chamber view (Panel c) and the parasternal short axis view (Panel d)

In the IDM with suspected HCM, NPE is utilized to evaluate ventricular dimensions (size, area, volume) and function, the severity of dynamic LVOT obstruction, the presence of mitral valve abnormalities; special attention is paid towards the septum and its relationship to the LVOT. Both M-mode and 2D echocardiography are utilized. Linear internal measurements of the LV are acquired in the parasternal long-axis view. Volume measurements are based on tracings of the blood-tissue interface in the apical four- and two- chamber views. In the normally shaped LV, both M-mode and 2D echocardiographic formulas are used to calculate LV mass, of which normal reference ranges exist for term and preterm infants.^79^ The interventricular septum is best visualized from the parasternal long axis view and short axis view (Fig. 2a,b). The degree of LVOT obstruction and the relationship of the septal hypertrophy to the mitral valve leaflets must be assessed from the parasternal long axis view. The measurement of systemic and pulmonary blood flow should be assessed, but during the transitional period it can be complicated by the presence of ductal and atrial shunting. Left ventricular output (LVO), the usual measure of cardiac output, is not representative of systemic blood flow when the PDA is open, as LVO is measured before the ductus and therefore includes both systemic blood flow and the ductal contribution to pulmonary blood flow. RV output (RVO) measures systemic venous return (systemic blood flow) plus left to right atrial shunting.^80^ Increased left to right atrial flow is an indirect measure of increased pulmonary blood flow. Superior vena cava (SVC) flow is not affected by the PDA or FO shunts, which makes it a proxy measure of systemic blood flow during early transition. It should be noted that LVO and RVO calculations may be inaccurate in the presence of non-laminar flow due to mid-cavity or outflow tract obstruction with flow acceleration. Screening for mid-cavity flow acceleration may be warranted. RV performance (structure and function) is measured from the apical view and the parasternal long axis view to properly assess inflow and outflow linear dimensions. RV areas are assessed during end-diastole and end-systole from the apical four-chamber view.

#### Advances in echocardiographic techniques

Normal strain (longitudinal) and shear strain (rotational mechanics in the circumferential-longitudinal plane) have recently been studied in IDMs.^81–83^ Al-Bitalgi showed that torsion and LV longitudinal systolic strain by STE were significantly impaired in IDMs during the transitional period.^81^ Liao et al. demonstrated a similar decrease in torsion, but with preserved EF, suggesting that rotational mechanics may offer a more sensitive measure of ventricular function.^82^ Systolic function is thought to be preserved in IDM, yet the use of more sensitive echocardiographic techniques have revealed that systolic dysfunction may be evident at birth and persist through 1 month of age.^81,83^ Cade et al. showed that septal wall thickness was not different at 1 month, but global and segmental systolic and diastolic strain values were significantly lower in IDM.^83^ Therapeutic approaches are beyond the scope of this review but should ensure adequate LV filling (maintenance of preload) and avoidance of diuretics which can considerable worsen condition (by compromising LV filling). Maintaining adequate afterload (to minimize the pressure gradient across the LVOT) and avoiding tachycardia are the other two mainstays of treatment.

### Myocarditis

Myocarditis is an inflammatory process that affects the heart muscle and can cause rare, yet devastating HF in neonates.^84^ Myocarditis has been classified as both an inflammatory and infectious cause of cardiomyopathies, which is reflective of its myriad presentations range from minimal symptoms to severe HF and sudden death.^4^ Myocarditis accounts for approximately 15% of patients with DCM in children, but is less common in neonates (although it may be under-reported; ^12^). Although the incidence in neonates is decreasing, it remains a significant complication of infection.^85^ Neonatal infectious myocarditis can be caused by several viruses (Coxsackie B, parvovirus, herpes simplex, and adenovirus) or bacteria (group B streptococcal), but is predominantly caused by Coxsackie B. Neonatal Coxsackie B myocarditis can affect both full-term and preterm infants and usually presents within the first week of age.^85^ The signs of neonatal myocarditis are nonspecific and range from mild respiratory or gastrointestinal illness to significant meningoencephalitis.^86^ Manifestations include congestive HF, tachycardia, and electrocardiographic changes.^85^

In neonates, cardiac muscle involvement is more prominent and the myocardial damage primarily occurs due to direct lysis of infected myocytes during the initial viral infection phase.^87^ In the second stage, the inflammatory phase, activation of the host immune system by the primary viral infection causes myocellular damage that results in myocardial dysfunction (often LV) leading to impaired contractility and HF.^88^ The acute myocarditis is commonly followed by complete recovery of LV function, but can progress to a secondary DCM with chronic HF.^84^ During this third phase, some neonates will develop significant arrhythmias (atrial or ventricular), conduction disturbances, or circulatory collapse.^84,85^ This cardiovascular collapse may occur several days after the onset of the sepsis-like illness during this second wave of a biphasic pattern of infection.^87,88^ The prognosis for neonates that develop myocarditis varies. Neonatal myocarditis can be fatal in early infection. However, for patients that survive the acute illness, recovery with few long-term sequelae may be seen. Full recovery can be prolonged and associated arrhythmias may persist for months afterwards.

#### Cardiovascular assessment of a neonate with myocarditis

The diversity of signs, symptoms, and clinical presentations accompanying acute myocarditis makes its diagnosis challenging. The clinical symptoms encountered in neonates may include tachycardia, tachypnea with abnormal respiratory patterns, cyanosis, and poor perfusion. Neonates may not tolerate feedings and present with emesis. Fever (or hypothermia) may be present. Other cardiac specific signs such as pallor, hypotension, edema, and hepatomegaly occur in only a minority of cases.^84^ The methodical approach to neonates with suspected myocarditis will account for the varied differentials that range from bacterial/viral sepsis, critical congenital heart diseases with ductal dependent systemic circulation to acute metabolic crises.^87,88^ Routine laboratory investigations should also include cardiac enzyme levels (e.g. cardiac troponins). Electrocardiogram (ECG) findings are usually nonspecific and include sinus tachycardia, PR prolongation and nonspecific ST changes. ST segment and T-wave abnormalities are the typical ECG patterns, but they are usually transient.^89^ Cardiorespiratory focused therapies (intubation, ventilation, inotropic support and/or ECMO) may be indicated based on the level of support appropriate for the clinical symptoms, but the specifics are beyond the scope of this review. The prognosis is poor and further aggravated by prematurity.^90^

#### Clinical indications for NPE

NPE should be performed when myocarditis is suspected in a neonate with new onset clinical findings of cardiac dysfunction (especially following a viral prodrome in the mother, and in particular in neonates without an underlying structural cardiac defect), a rise in cardiac biomarkers, or ECG changes suggestive of acute myocardial injury or arrhythmia.

#### Echocardiographic assessment techniques

The gold standard for diagnosing myocarditis is endomyocardial biopsy, however the invasive nature and potential serious complications, make this impractical in neonates. Non-invasive techniques, such as echocardiography, to detect myocarditis are necessary. Traditional echocardiography findings are LV regional or global dysfunction, LV wall motion abnormalities, and LV dilatation. However, some studies in adults with histologically proven myocarditis have also found evidence of RV dysfunction,^91^ and others have shown RV dysfunction to be an independent predictor of outcome.^92^ In addition, the presence of myocardial interstitial edema and pericardial effusions (myopericarditis) in acute myocarditis leads to thickening of the ventricular wall, which can also be detected by echocardiography. Although the most common echocardiographic finding associated with myocarditis is a DCM phenotype, hypertrophic and restrictive phenotypes have been described in histologically proven myocarditis in adults.^91^ Fulminant myocarditis is less common in neonates, but has a distinct symptom complex from acute myocarditis with characteristic echocardiographic phenotype of reduced LV ejection, normal cavity size, and increased septal thickening.^93^

#### Advances in echocardiographic techniques

Although conventional echocardiography is considered as a reliable tool for ventricular wall motion evaluation, the visual estimation is subjective and has high inter-observer and intra-observer variability in neonates. There is growing evidence from studies in adult patients that has evolved from case reports to prospective studies that utilizes deformation imaging techniques to asses ventricular function in patients with acute myocarditis at risk to develop DCM.^89,94^ In children and neonates with myocarditis there are a paucity of reports on the utility of these novel echocardiographic modalities.^95^ Although the literature is expanding, most of the adult studies lack a correlation to muscle biopsy or even cardiovascular magnetic resonance imaging, limiting the reliability of the measures in this clinical scenario. Nonetheless, the findings suggest there may be a role for using deformation imaging in the diagnosis and management of acute myocarditis.

### Arrhythmia-induced neonatal cardiomyopathy (AINC)

Fetal and early persistent neonatal arrhythmias can cause a transient DCM phenomenon, referred to as arrhythmia-induced neonatal cardiomyopathy (AINC). In neonates, all tachyarrhythmia (even supraventricular and ventricular ectopy) could potentially cause AINC,^96^ but atrial ectopic tachycardia (AET) is the most common of AINC.^97^ In older infants and children, the etiologies of cardiomyopathy also include re-entry tachycardia (i.e. WPW) and permanent junctional reciprocating tachycardia (PJRT; ^98^). Tachycardia-induced cardiomyopathy (also referred to as tachycardia-mediated cardiomyopathy and tachymyopathy) is a rare entity of AINC caused by prolonged periods of sustained tachycardia. Neonatal complete heart block seen in association with a maternal autoimmune disease or a congenital cardiac malformation can rarely lead to AINC. The cardiomyopathy and accompanied LV systolic dysfunction is transient, often normalizing following treatment to restore normal sinus rhythm, and carries a favorable prognosis in younger infants.^98^ The tachycardia could either be the primary cause of the cardiomyopathy, or secondary to a cardiomyopathy of different etiology.

The incidence of AINC is difficult to estimate due to a paucity of published case reports in neonates and case series in children. This is probably an under-appreciated etiology of dilated cardiomyopathy and myocardial dysfunction in children.^96^ The sustained tachycardia in AINC may lead to abnormal cellular remodeling, diminishing the number of microtubules in the cardiac myocyte and contributing to contractile dysfunction.^99^ Chronic tachycardia may lead to a depletion of high-energy phosphates and cellular reduction in sarcolemmal sodium/potassium ATPase activity and enzyme distribution that may affect calcium handling.^100^ Neonates will therefore have more rapid deterioration in function due to lower intracellular calcium reserves.^96^

#### Cardiovascular Assessment of a Neonate with AINC

The clinical presentation of AINC is variable in neonates and although a high index of clinical suspicion may point to subtle diagnostic clues, the condition may still go unrecognized initially.^99^ The first presenting sign may either be the arrhythmia or the clinical symptoms that manifest as systolic HF develops. Even a single, sustained episode of typical supraventricular tachycardia may be unrecognized until HF symptoms emerge; thus, neonates may present with clinical signs of shock.^96,99^ If the arrhythmia is first recognized after birth, the infant may still be asymptomatic, but left untreated or unrecognized after a few days to weeks of accelerated ventricular rates, signs of congestive heart failure and a state of cardiac contractile dysfunction can evolve because of the DCM.^96^ AINC can also present with tachycardia interspersed with periods of sinus rhythm, and therefore the responsible tachycardia may also not be evident at presentation.^96,97^ The neonate cannot verbalize the common symptoms of palpitations, further complicating the early identification of heart failure. Neonates with initial symptomatic AINC might have also had fetal tachycardia.

The diagnostic evaluation of AINC initially relies on electrocardiogram (ECG) to document the cardiac rhythm and ventricular heart rate. Comparisons to prior ECGs should be routinely performed, when available. Usually the diagnosis of AINC can only be made following a successful trial of therapy to slow the ventricular rate or to restore sinus rhythm along with the exclusion of other potential causes of cardiomyopathy.^99^ Therapy options are beyond the scope of this review, but should be tailored to the suspected arrhythmia that is inducing the cardiomyopathy.^99^ In contrast to adult cases of AIC, where LV systolic functional recovery may take weeks to months and reverse remodeling may take years to bring the heart rate and rhythm under control, in neonate the median time to recovery is reported at less than 2 months.^98,99^

#### Clinical indications for NPE with neonatal arrhythmia

A high index of clinical suspicion of AINC should be suspected in neonates with a history of fetal tachycardia and evidence of new onset LV systolic dysfunction soon after birth. These infants should undergo NPE to assess cardiac structure and function.

#### Echocardiographic assessment techniques

Systolic ventricular dysfunction is often the first manifestation of AINC, followed by LV dilatation after prolonged dysfunction (Fig. 2c,d; ^101^). Neonates with AINC will have a smaller LV end-diastolic diameter and smaller LV Mass index than those with pre-existing dilated cardiomyopathy and concomitant tachyarrhythmia. In addition, LV end-systolic dimensions, EF, and the degree of mitral regurgitation should be assessed.^98^

#### Advances in echocardiographic techniques

There are no reported studies in the literature that utilize emerging measures to characterize cardiac performance in neonates with AINC.

## Conclusion

Neonatal HF is a clinical condition that results from impairment of the ventricle to fill with or eject blood. The causes of HF can be divided into pathophysiologic categories that aid in the understanding of the underlying physiology and clinical manifestations (see Table 4). Neonatal cardiomyopathy is the most common cause of heart failure in a neonate with a structurally normal heart. A high index of suspicion of cardiomyopathy based on the common identifiable perinatal etiologies is critical in guiding the approach to management. Cardiomyopathies are disorders of the myocardium that result in alterations of cardiac chamber size and resultant systolic and diastolic dysfunction, with DCM and HCM as the two most common phenotypes in neonates. Echocardiography is the most used, efficient, and accessible technique for establishment of the diagnosis of cardiomyopathy in neonate. The integration of echocardiographic features with clinical and laboratory findings will allow for a better assessment of immediate risk and long-term prognosis in neonates with a cardiomyopathy.

**Table 4:** Summary of recommendations regarding the use of NPE in newborns with heart failure unrelated to congenital structural heart disease

**PERINATAL HYPOXIC ISCHAEMIA**
Early comprehensive NPE is indicated in neonates who suffer from a perinatal hypoxic ischemia event and have clinical or biochemical signs of cardiovascular compromise.
If signs of LV dysfunction are apparent, a structural echocardiogram must be performed to evaluate for normality, with a focus on the coronary arteries.
Standard NPE, including the assessment of LV and RV function, PPHN, and ductal shunting, provides additional information to identify when there is significant cardiovascular impairment, classify the underlying abnormal physiology and potentially target appropriate therapy.
Combined with the clinical examination and serum biomarkers, NPE will permit rapid and accurate diagnosis, allow for early initiation and monitoring of therapy, and provide longitudinal assessment of hemodynamic function and cardiac performance during the cooling and rewarming phases of TH.
For those neonates requiring significant cardiopulmonary support, careful functional monitoring during each phase may be warranted.
Future research needs to focus on diastolic heart function, the relationship between poor myocardial performance and acute brain injury, the effects of cardiovascular intervention during the cooling and rewarming phases, and long-term cardiovascular outcomes in relation to neurodevelopmental status.
**DIABETIC CARDIOMYOPATHY**
There must be a high index of suspicion for HCM in IDM patients.
The NPE assessment of an IDM with suspected HCM should include evaluation of ventricular dimensions (size, area, volume) and function, with special attention paid towards the septum and its relationship to the LVOT. Both M-mode and 2D echocardiography are utilized.
Deformation imaging and rotational mechanics may offer additional functional information, but they should be utilized as adjunct modalities until further data is available.
**MYOCARDITIS**
NPE should be considered in any neonate that presents with sepsis-like symptoms and clinical signs of LV dysfunction, especially in the setting of a known maternal viral prodrome. These infants should be serially monitored for DCM, arrhythmias, and potential circulatory collapse that may develop during the 3^rd^ stage of the viral myocarditis.
**ARRHYTHMIA-INDUCED NEONATAL CARDIOMYOPATHY**
AINC is a rare, often transient cause of DCM in neonates. A high index of suspicion is necessary to properly diagnose the HF in neonates at risk for AINC.
If there is long-standing fetal arrhythmia, new onset LV dysfunction without neonatal arrhythmia (and structurally normal heart), or sustained postnatal arrhythmia, we suggest utilizing NPE to provide detailed structural and functional analysis.
There is a predictable pattern of resolution with treatment following arrhythmia control, and failure to recover should instigate a search for an underlying cardiomyopathy.

## Appendix

**European Special Interest Group ‘Neonatologist Performed Echocardiography’ (NPE), endorsed by the European Society for Paediatric Research (ESPR) and European Board of Neonatology (EBN)**

**de Boode W. P.** (chairman), Department of Neonatology, Radboud University Medical Center, Radboud Institute for Health Sciences, Amalia Children’s Hospital, Nijmegen, The Netherlands (willem.deboode@radboudumc.nl)

**Austin T.**, Department of Neonatology, Rosie Hospital, Cambridge University Hospitals NHS Foundation Trust, Cambridge, United Kingdom (topun.austin@addenbrookes.nhs.uk)

**Bohlin K.**, Department of Neonatology, Karolinska University Hospital, Karolinska Institutet, Stockholm, Sweden (kajsa.bohlin@ki.se)

**Bravo M. C.**, Department of Neonatology, La Paz University Hospital, Madrid, Spain (mcarmen.bravo@salud.madrid.org)

**Breatnach C. R.**, Department of Neonatology, The Rotunda Hospital, Dublin, Ireland (colm.breatnach@gmail.com)

**Breindahl M.**, Karolinska University Hospital, Karolinska Institutet, Stockholm, Sweden (morten.breindahl@sll.se)

**Dempsey E.**, INFANT Centre, Cork University Maternity Hospital, University College Cork, Ireland (g.dempsey@ucc.ie)

**El-Khuffash A.**, Department of Neonatology, The Rotunda Hospital, Dublin, Ireland; Department of Pediatrics, The Royal College of Surgeons in Ireland, Dublin, Ireland (afifelkhuffash@rcsi.ie)

**Groves A. M.**, Division of Newborn Medicine, Mount Sinai Kravis Children’s Hospital, New York, NY, USA (alan.groves@mssm.edu)

**Gupta S.**, University Hospital of North Tees, Durham University, Stockton-on-Tees, United Kingdom (samir.gupta@nth.nhs.uk)

**Horsberg Eriksen B.**, Department of Pediatrics, Møre and Romsdal Hospital Trust, Ålesund, Norway (beate.eriksen@me.com)

**Levy P. T.**, Department of Pediatrics, Washington University School of Medicine, Saint Louis, MO, USA; Department of Pediatrics, Goryeb Children’s Hospital, Morristown, NJ, USA (Levy_P@kids.wustl.edu)

**McNamara P. J.**, Departments of Pediatrics and Physiology, University of Toronto, Toronto, ON, Canada (patrick.mcnamara@sickkids.ca)

**Molnar Z.**, John Radcliffe Hospital, Oxford, United Kingdom (zoltan.Molnar@ouh.nhs.uk)

**Nestaas E.**, Institute of Clinical Medicine, Faculty of Medicine, University of Oslo, Norway; Department of Cardiology and Center for Cardiological Innovation, Oslo University Hospital, Rikshospitalet, Oslo, Norway; Department of Paediatrics, Vestfold Hospital Trust, Tønsberg, Norway (nestaas@hotmail.com)

**Rogerson S. R.**, The Royal Women's Hospital, Parkville, VIC, Australia (sheryle.Rogerson@thewomens.org.au)

**Roehr C. C.**, Department of Paediatrics, University of Oxford, John Radcliffe Hospital, Oxford, United Kingdom (charles.roehr@paediatrics.ox.ac.uk)

**Savoia M.**, Azienda Ospedaliero-Universitaria S. Maria della Misericordia, Udine, Italy (marilena.savoia@gmail.com)

**Schubert U.**, Department of Clinical Science, Intervention and Technology, Karolinska Institutet, Stockholm, Sweden (ulfschubert@gmx.de)

**Schwarz C. E.**, Department of Neonatology, University Children’s Hospital of Tübingen, Tübingen, Germany (c.schwarz@med.uni-tuebingen.de)

**Sehgal A.**, Department of Pediatrics, Monash University, Melbourne, Australia (arvind.sehgal@monash.edu)

**Singh Y.**, Addenbrooke's Hospital, Cambridge University Hospitals NHS Foundation Trust, Cambridge, United Kingdom (yogen.Singh@nhs.net)

**Slieker M. G.**, Department of Paediatric Cardiology, Radboudumc Amalia Children’s Hospital, Nijmegen, The Netherlands (Martijn.Slieker@radboudumc.nl)

**Tissot C.**, Department of Pediatrics, Clinique des Grangettes, Chêne Bougeries, Switzerland (cecile.tissot@hotmail.com)

**van der Lee R.**, Department of Neonatology, Radboud University Medical Center, Radboud Institute for Health Sciences, Amalia Children’s Hospital, Nijmegen, The Netherlands (Robin.vanderLee@radboudumc.nl)

**van Laere D.**, Department of Pediatrics, Antwerp University Hospital UZA, Edegem, Belgium (david.VanLaere@uza.be)

**van Overmeire B.**, Department of Neonatology, University Hospital Brussels, Brussels, Belgium (bart.van.overmeire@erasme.ulb.ac.be)

**van Wyk L.**, Department of Paediatrics & Child Health, University of Stellenbosch, Cape Town, South Africa (lizelle@sun.ac.za)
